# G-CSF Intrauterine for Thin Endometrium, and Pregnancy Outcome

**Published:** 2015-09

**Authors:** Ensieh Tehraninejad, Fateme Davari Tanha, Ebrahim Asadi, Koorosh Kamali, Elham Aziminikoo, Elahe Rezayof

**Affiliations:** 1 Reproductive Health Research Center, Tehran University of Medical Sciences, Tehran, Iran; 2 Department of Reproductive Endocrinology, Women’s Hospital, Tehran University of Medical Sciences, Tehran, Iran; 3 Department of Reproductive Biology, Women’s Hospital, Tehran University of Medical Sciences, Tehran, Iran; 4 Department of Public Health, School of Public Health, Zanjan University of Medical Sciences, Zanjan, Iran

**Keywords:** ART cycle, thin endometrium, G-CSF

## Abstract

**Objective:** To evaluate effects of G-CSF on a cancelled ART cycle due to thin endometrium.

**Materials and methods:** In a nonrandomized clinical trial from January 2011 to January 2013 in two tertiary university based hospitals fifteen patients undergoing embryo transfer and with the history of cycle cancellation due to thin endometrium were studied. Intrauterine infusion of G-CSF was done on the day of oocyte pick-up or 5 days before embryo transfer. The primary outcome to be measured was an endometrium thickened to at least 6 mm and the secondary outcome was clinical pregnancy rate and consequently take-home baby. All previous cycles were considered as control for each patient.

**Results:** The G-CSF was infused at the day of oocyte retrieval or 5 days before embryo transfer. The endometrial thickness reached from 3.593±0.251 mm to 7.120 ± 0.84 mm. The mean age, gravidity, parity, and FSH were 35.13± 9.531 years, 3, 1 and 32.78 ± 31.10 mIU/ml, respectively. The clinical pregnancy rate was 20%, and there was one missed abortion, a mother death at 34 weeks, and a preterm labor at 30 weeks due to PROM.

**Conclusion:** G**-**CSF may increase endometrial thickness in the small group of patients who had no choice except cycle cancellation or surrogacy.

## Introduction

During implantation, blastocyst attach to endometrium in secretory phase (1). The relationship between the developing embryo and maternal tissue plays an important role in Successful implantation (2). Studies show that positional injury in endometrium can increase the rate of implantation and pregnancy due to enormous release of growth factors and cytokines from the site of injury (3, 4). G-CSF plays an important role in human reproductive achievement. It is believed that G-CSF has important function in follicular maturation, ovulation, implantation and pregnancy after conception, uterine epithelial cell G-CSF expression remains high for the first few days then due to inhibitory effect of progesterone its levels declines around the time of embryo implantation. In addition, G-CSF concentration in endometrial co-culture correlated with pregnancy luck (5).

The level of G-CSF in serum and follicular fluid is a prophesier for the success of human IVF (6). The normal thickness of endometrium is 7 to 14 mm in the secretory phase (7, 8) and it is a prominent factor for successful pregnancy in IVF cycles (9, 10). 

Studies show that pregnancy will not happen if endometrium thickness is less than 6mm (11, 12).

Furthermore, thin endometrium causes higher risk of miscarriage (13). One obvious potential explanation for the effects of G-CSF deficiency on pregnancy outcome is a role in targeting leukocytes required for establishing or maintaining pregnancy. Traditional treatment such as low dose aspirin, vaginal sildenafil, pentoxifylline, tocopherol, and estrogen administration are widely inefficient (14- 16). Only 0.6%-0.8% patients do not reach to the minimum thickness of the endometrium (17). Moreover, clinician suggest that the IVF cycle should be canceled and all the embryos should be cryopreservated, however, in future cycles there is merely a probability of reaching to the sufficient endometrium thickness. In the other way, clinician would transfer the embryo even though; the chance of pregnancy might decrease. In the present survey, we describe the impact of G-CSF infusion on unresponsive thin endometrium and ART outcome.

## Materials and methods

This study was approved by the ethical committee of deputy research of Tehran University of Medical Sciences and was registered in (Iranian registry for clinical trial) IRCT by number: IRCT201012272576N4 and informed consent had been obtained from all patients.

Fifteen infertile women have been included. The inclusion criteria were history of IVF cycle cancellation due to thin endometrium. The age of patients ranged from 22 to 45 years old and their characteristics are summarized in the table-1. They were selected from the patients who referred to our clinic for ART cycle from June 2011 to September 2012. Their ART cycle had been canceled due to thin endometrial thickness (ET < 6mm) whereas routine treatments are administrated such as estradiol 2mg twice daily orally, 1mg three times daily per vagina and sildenafil citrate vaginal suppositories, 25 mg four times daily.

At first, ovaries were stimulated with a standard protocol (GnRH Agonist /GONAL-f ®, Merck Serono, Germany) or GnRH Antagonist /GONAL-f). When at least two follicles achieved 18mm diameter, Human chorionic gonadotropin (Ovitrelle 250 micrograms/0.5 ml, Merck Serono, Germany) was administered for ovulation triggering. Transvaginal oocyte retrieval was performed at 36 hrs after injection of hCG. The oocytes were fertilized by intra cytoplasmic sperm injection (ICSI) method. The thickness of endometrium was evaluated on the day of oocyte retrieval and a syringe which containing (300mcg/1ml) G-CSF (Neupogen^TM^ , Filgastrim, Amgen Inc., Thousand Oaks, Canada) Was infused slowly in the uterine cavity and after 5 days endometrial thickness was evaluated again.

To judge implantation, the levels of serum β-hCG were measured 16 days after embryo transfer. The clinical pregnancy was confirmed by ultrasound observation of pregnancy sac. Ultrasound assessment was used for measuring thickness of the endometrium before and after G-CSF infusion. All scans were done by one operator, with a Siemens elegra and a 6.5 MHz transvaginal probe. Endometrial thickness was measured in the sagital plane. It was described as the minimal distance from the outer edge of endometrial myometrial interface to the outer edge in the widest part of the endometrium. All of these patients had at least one cycle which was cancelled because of thin endometrium and that cycle was considered as control cycle for each patient, which means that every patient was her own control in prior cycle.The paired t-test was used to evaluate the change in the thickness of endometrium before and after G-CSF infusion and the Mann-Whitney u-test was employed to compared the differences between thickness of endometrium and other individuality of patients who became pregnant or not. The statistical analysis was done using SPSS version 16, the significance was defined as p ≤ 0.05.

## Results

Fifteen patients undergoing intrauterine G-CSF infusion before embryo transfer who had thin endometrium regardless of treatment with oral and vaginal esteradiol and sildnafil. Demographic characteristic of patients are presented in table-1.

All patients had thin endometrium (mean = 3.6 ± 0.98) at the time of infusion, after intrauterine infusion of G-CSF mean of endometrial thickness was 7.12 ± 0.84 (p value < 0.001). 

**Table 1:** Demographic characteristics of patients (n= 15)

Age (year) (mean ± SD)	35.13 ± 9.531	
Parity (mean)		1
Gravidity (mean)		3
Failed prior IVF cycles (n) (mean ± SD)		1.2 ± 0.532
FSH on cycle day 3 (mIU/ml) (mean ± SD)		32.78 ± 31.10
Cause of infertility		
Diminished ovarian reserve [n (%)]		7 (46.6%)
Male factor [n (%)]		2 (13.3%)
Uterine factor [n (%)]		4 (26.7%)
PCO [n (%)]		2 (13.3%)
Duration of infertility (year) (mean ± SD)		11.20 ± 7.73

Two patients had history of tubercoulosis endometritis which had been received standard treatment for tuberculosis and after medical treatment hysteroscopy and adheisolysis was done. They had very resistant thin endometrium (3mm) who had 2 times cycle cancellation. After G-CSF infusion endometrial thickness (ET) was 5mm and they referred for surrogacy.

Four other patients had infertility due to uterine factor and adheisionolysis was done with hysteroscopy before ICSI cycles, all these four had increased endometrial thickness after intranuterine infusion of G-CSF, but just one of them got pregnant.

We transferred 2-3 good quality embryos for 13 patients except two who had endometrial thickness less than 6 mm, with history of endometrial tuberculosis. The clinical pregnancy rate was 20%.

Table 2 presents endometrial thickness in patients before and after treatment with G-CSF. 

There was significant difference between endometrial thickness at the time of G-CSF infusion versus day of embryo transfer in cycle leading to pregnancy (p = 0.002) and those not resulting in pregnancy (p = 0.04).

Table 3 demonstrates outcome in women who conceived, there were three pregnancies. The first patient got pregnant and pregnancy continued until 34 weeks, which mother had died due to gas (CO) toxicities in her home.

The second patient got pregnant but at 12 weeks, pregnancy was terminated due to missed abortion.

The third patient who got pregnant terminated with cesarean delivery at 30 weeks due to premature rupture of membrane and the neonatal weight was 1800 gr.

Table 4 shows comparison between endometrial thickness at past cancelled cycle at the day of OPU and at the cancellation day, however the mean of endometrial thickness difference was statistically significant, because the final thickness was 5.18±0.41 the cycles were cancelled. 

The Δ difference between endometrial thickness before and after intrauterine infusion of G-CSF was 3.53 ± 0.88. Also the Δ difference between past cycle and GCSF cycle was 1.36 ± 1.1, p value = 0.001.

## Discussion

In this study, we found that granulocyte- colony stimulating factor may improve endometrial thickness from 3.3 to 6.1mm. Furthermore, it seems that this effect was associated with a higher potential of pregnancy.

**Table 2:** Endometrial thickness in patients before and after treatment with G-CSF (n = 15)

		**Conceived** **(n = 3)**	**Not conceived** **(n = 12)**
Endometrial thickness at day of GCSF infusion (mean ± SD)	3.6mm ± 0.98	3.6mm ± 1.5	3.4mm ± 0.87
Endometrial thickness at day of embryo transfer (mean ± SD)	7.120mm ± 0.84	7.5mm ± 1.4	7.mm ± 0.71
Δ endometrial thickness (mean± SD)	3.53mm ± 0.88	4.2mm ± 1.3^a^	3.6mm ± 0.72^b^

a Wilcoxon test p value = 0.109;

b Wilcoxon test p value = 0.002

**Table 3:** Patients’ outcome in case of pregnancy

**Patient **	**age**	**Outcome**	**Diagnosis**	**Times of cycle ** **cancellation due to thin ** **endometrium**	**Day of ** **GCSF ** **infusion**	**Endometrium ** **before/after ** **(mm)**	
1	36	Mother died at 3th trimester a	Uterine factor	1	OPU	3	7.6
2	30	Missed abortion	POF	1	OPU	5	7.9
3	45	Preterm labor 30w	Old age	1	5 days before ET	4	8.1

a due to CO toxicity

**Table 4:** Endometrial thickness in previous cancelled cycles and current cycle (G-CSF cycle)

	**OPU day**	**cancellation ** **day**	**Difference** **(mean ± SD)**	**Embryo ** **transfer (n)**	**Clinical ** **pregnancy ** **[n (%)]**	**Number of cancelled ** **patients [n (%)]**
Previous cycle	3.01± 0.5	5.18 ± 0.41	2.17 ± 0.74	0	0 (0%)	15 (100%)
Current cycle	3.6 ± 0.98	7.120 ± 0.84	3.53 ± 0.88^a^	13	3 (20%)	2 (13.3%)

a p value = 0.001

Actually, G-CSF may increase the thickness of endometrium in the patient with extremely thin endometrium when other methods to improve endometrial thickness are widely inadequate. Another treatments such as low dose aspirin and vaginal sildenafil increase blood supply of uterine rather than expand the thickness of endometrium (14, 15). Alternative treatment like tocopherol and pentoxifylline increase endometrial thickness and pregnancy outcome but length of treatment period ought to be 6-9 months (16). In our study, we used G-CSF in patients who have thin endometrium in order to avoid canceling their ART cycle. In fact, increasing the pregnancy success was not our study’s aim although; our data show that the chance of pregnancy may increase because of improving endometrial thickness after administering of G-CSF. In addition, other studies suggested that granulocyte- colony stimulating factor increase implantation rate in human and animal model (18, 19) and G-CSF receptors that are found in the trophoblast and decidua are necessary for implantation (20, 21).The effect of G-CSF and their receptors in implantation may play an important role in increasing the rate of pregnancy. Another author have reported that Patients with a good response to ovarian stimulating protocol (rFSH) demonstrated high level of G-CSF in serum and follicular fluid compared with patients who had low response to ovarian stimulating with rFSH. In addition, the pregnancy rate of the first group was 33.5% whereas pregnancy did not occur in the second group (6). Also others show that G-CSF is a member of implantation window as a result of its concentration at the time of implantation (6). These reports are similar to our findings and support them in association with improving endometrial thickness and increasing the possibility of success in ART cycle. Esteradiol and sildnafile induce averagely 3.01 mm increment in the endometrial thickness, although the final endometrial thickness was < 6.5 mm and all cycle had to be cancelled. But G-CSF increases the endometrial thickness averagely 4.106mm and in comparison to esteradiol and sildnafil alone it increases 1.36mm more thickening, so the final endometrial thickness was more suitable (7.12 ± 0.218) for embryo transfer. 

It seems that G-CSF may have a role for increasing thickness in association with esteradiol and sildnafil and it may be a useful option in thin endometrium refractory to the other treatments.

However others report that in 3 cases G-CSF couldn’t improve thin endometrium to a more fertile level and all failed to conceive in G-CSF cycle but one of them conceived on another cycle without G-CSF and surprisingly with endometrial thickness 4mm (22), the sample size is lower than current study.

In two another study there was remarkable results (23, 24) ,in first study four case with refractory thin endometrium had intrauterine infusion of G-CSF and all four cases had found endometrial thickness suitable for embryo transfer, one twin pregnancy and two singleton and one intramural ectopic pregnancy which had terminated, but the results of two other was reported as ongoing pregnancy at the time of article publication, in current study there were three pregnancy that just one of them results in take home baby and unfortunately one pregnant mother died at third trimester and one of them had missed abortion at first trimester, and current study is unique because followed the pregnant women until end of pregnancy, however the results are not as remarkable as the first study by Gleicher, still the result are noticeable, and the sample size was more (23).

In the second study by Gleicher in 21 patients with thin endometrium the ongoing clinical pregnancy rate was 19.1%, like the present study, but our study reports the results at the end of study (24). 

The limitation of current study is the small sample size, and nonrandomized method, due to limited number of patients. We suggest a randomized clinical trial with greater sample size for evaluating the effect of G-CSF on thin endometrium.

## Conclusion

Infusion of G-CSF in endometrial cavity is a safe and probably effective method to increasing endometrial thickness for patients with thin and unresponsive endometrium.
